# Next‐generation phylogeography of the cockle *Cerastoderma glaucum*: Highly heterogeneous genetic differentiation in a lagoon species

**DOI:** 10.1002/ece3.5070

**Published:** 2019-03-27

**Authors:** Ludmila Sromek, Didier Forcioli, Rafal Lasota, Paola Furla, Maciej Wolowicz

**Affiliations:** ^1^ Department of Marine Ecosystems Functioning, Institute of Oceanography, Faculty of Oceanography and Geography University of Gdansk Gdynia Poland; ^2^ UPMC Université Paris 06, Université Antilles, Université Nice Sophia Antipolis, CNRS, Laboratoire Evolution Paris Seine, Institut de Biologie Paris Seine (EPS‐IBPS) Sorbonne Universités Paris France

**Keywords:** *Cerastoderma glaucum*, coastal lagoons, mito‐nuclear discordances, outlier loci, phylogeography, postglacial range expansion, RADseq

## Abstract

**Aim:**

Coastal lagoons form an intriguing example of fragmented marine habitats. Restricted gene flow among isolated populations of lagoon species may promote their genetic divergence and may thus provide a first step toward speciation. In the present study, the population genetic structure of the lagoon cockle *Cerastoderma glaucum *has been investigated to clarify the complex phylogeographic pattern found in previous studies, to localize major genetic breaks, and to discuss their origin and maintenance.

**Location:**

The Atlantic and Mediterranean coasts, including the Baltic, North Sea, and Black Sea.

**Methods:**

A total of 204 *C. glaucum* individuals from 14 populations were genotyped using restriction site‐associated DNA sequencing (RADseq). The genetic diversity, divergence, and structure were analyzed using genome‐wide single nucleotide polymorphisms (SNPs). Phylogenetic relationships were inferred under a coalescent model using svdquartets.

**Results:**

The RADseq approach allowed inferring phylogeographic relationships with an unprecedented resolution. Three deeply divergent lineages were identified within *C. glaucum *that are separated by many genetic barriers: one lineage in the Aegean–Black Sea region, one in the Ionian Sea, and the last one widely distributed from the Western Mediterranean to the Baltic Sea. The nested branching pattern displayed on the species tree largely agrees with the likely scenario of *C. glaucum* postglacial expansion from the Mediterranean to the Baltic Sea.

**Main conclusion:**

The genetic differentiations between geographically separated lagoons proved to be strong, highlighting the evolutionary influence of these naturally fragmented habitats. The postglacial expansion created complex patterns of spatial segregation of genetic diversity with allele frequency gradients in many outlier loci, but also discrepancies between the nuclear and mitochondrial genetic markers that probably arose from genetic surfing of mitochondrial variation.

## 1 INTRODUCTION

Coastal lagoons form an example of fragmented marine habitat with discontinuous, patchy spatial distribution. Populations of lagoon species can be separated at a variety of spatial scales and differ in the mode and intensity of connection. A stepping‐stone model of population structure where populations exchange migrants along a one‐dimension spatial gradient (Kimura & Weiss, 1964) should particularly fit to this coastal habitat. A single colonization event may bring few founding individuals from one neighboring region to another, with no subsequent connection for hundreds of generations. However, within a given lagoon system, an island model (Wright, 1940) might also be applied, as many migrants may be exchanged between local subpopulations every generation. Moreover, populations of lagoon species have to cope with drastic environmental changes that frequently result in reduction of population size or even extinction. Thus, for some of the less resilient organisms, at a local scale, the lagoon habitats fit as well the metapopulation concept with its repeated extinction and recolonization events (Smedbol, McPherson, Hansen, & Kenchington, 2002). Restricted gene flow among isolated lagoon populations may facilitate not only genetic drift, but also local adaptation, as lagoon species face a wide variation of environmental parameters such as temperature and salinity (Bamber, Battens, Sheader, & Bridgwater, 1992). These isolated populations may respond independently to local selective regimes, thus adding to their genetic divergence. Overlapping patterns of morphometric and genetic differentiation between different Mediterranean lagoons have been found in the Mediterranean goby *Pomatoschistus tortonesei* (Mejri, Lo Brutto, Hassine, Arculeo, & Ben Hassine, 2012) and lagoonal sand smelt *Atherina lagunae* (Trabelsi, Maamouri, Quignard, Boussaïd, & Faure, 2004).

Large‐scale genetic structures of lagoon species reflect not only contemporary evolutionary processes linked to the heterogeneity in environmental conditions, but also species history (e.g., Pleistocene glaciations). For instance, Mejri, Arculeo, Ben Hassine, and Lo Brutto (2011) proposed that the complex genetic structure of the marbled goby *Pomatoschistus marmoratus* was shaped by past recurring shifts in sea level and water temperature of the Mediterranean Sea, which caused repeated recolonization events of the lagoon populations. A recent genome‐wide study of anchovy (*Engraulis encrasicolus*) revealed that two ecotypes (lagoon vs. offshore) probably evolved during a long period of allopatric isolation, and at present day, they are partially isolated, with semipermeable gene pools interacting with each other (Le Moan, Gagnaire, & Bonhomme, 2016). From this point of view, the population genetic structure of lagoon species may be regarded to be of fundamental interest for the understanding of the evolutionary mechanisms of population differentiation and, ultimately, of speciation (Cognetti & Maltagliati, 2000).

On the other hand, such complex pattern and recent phylogeographic events are not easily deciphered and require high‐resolution genetic markers to get the required information, and to compensate for the heterogeneity of genetic variation across the genome (Bierne, Gagnaire, & David, 2013; Landguth et al., 2012; Roux, Tsagkogeorga, Bierne, & Galtier, 2013). Genes under divergent selection and loci linked to such genes, as well as genes involved in genetic incompatibilities, are expected to show elevated genetic differentiation compared to the genomic average (Beaumont & Balding, 2004; Maynard Smith & Haigh, 1974; Nosil, Funk, & Ortiz‐Barrientos, 2009). Additionally, population structure derived from loci under selection may not follow genealogical relationships and current gene flow (Keller et al., 2013; Nadeau et al., 2013). Investigations of the relative roles of different evolutionary forces in shaping the distribution of genetic diversity are becoming easier in nonmodel organisms thanks to the advent of next‐generation sequencing technologies (McCormack, Hird, Zellmer, Carstens, & Brumfield, 2013). For example, the thousands of SNP markers obtained from the restriction site‐associated DNA (RAD) sequencing (Baird et al., 2008) display the results of evolutionary processes acting across the whole genome, rather than on the few loci analyzed in studies based on classical markers (Li et al., 2012; Narum, Buerkle, Davey, Miller, & Hohenlohe, 2013). The RADseq approach has been successfully used to investigate various questions in molecular ecology, for example, the heterogeneity of genomic divergence between different species or ecotypes (Hess, Campbell, Close, Docker, & Narum, 2013; Keller et al., 2013; Le Moan et al., 2016), fine‐scale population structure and recent colonization history (Catchen et al., 2013; Emerson et al., 2010; Jeffries et al., 2016), and interspecific phylogeny (Ebel et al., 2015; Herrera & Shank, 2016; Wagner et al., 2013).

The lagoon cockle *Cerastoderma glaucum *(Bruguière, 1789) is widely distributed from the Atlantic coast of Norway to the Mediterranean Sea (Boyden & Russell, 1972; Derbali, Hadj Taieb, Kammoun, Jarboui, & Ghorbel, 2014; Machado & Costa, 1994; Nicolaidou, Reizopoulou, Koutsoubas, Orfanidis, & Kevrekidis, 2005), including the Baltic Sea (Brock, 1991), North Sea (Reise, 2003), and Black Sea (David & Ţigan, 2011). It usually does not colonize open shores but rather thrives in nontidal lagoons, shallow creeks, ponds, and salt marshes, or more rarely on lower shores in estuaries. It tolerates salinities ranging from 5 to 60 PSU (Kingston, 1974). The failure of the species to colonize the open shores is believed to be due to an inability to tolerate loose sediment, air exposure, and wave action (Boyden & Russell, 1972; Brock, 1979). For these reasons, this species is considered as a lagoon specialist (Bamber et al., 1992). Apart from living in discrete lagoon habitats, the pelagic larval stage of *C. glaucum* lasts only about 1 week (Boyden & Russell, 1972); thus, a strong genetic structure is expected in this species.

**Figure 1:**
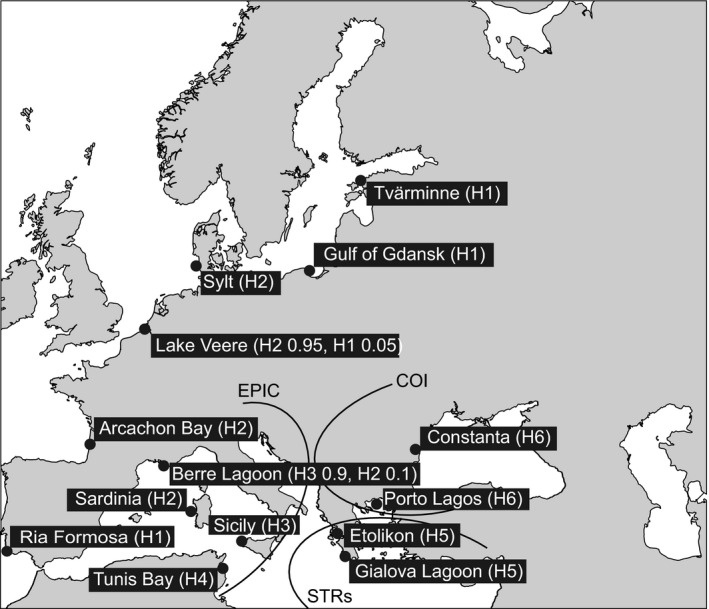
Sampling site locations and the main phylogeographic breaks described in previous studies of *Cerastoderma glaucum* based on COI, STRs, and EPIC markers (Sromek et al., 2016; Tarnowska et al., 2010). Numbers in parentheses indicate proportion of mitochondrial DNA haplogroups (arbitrarily numbered from H1 to H6) found in each sampling site by Tarnowska et al. (2010)

At present day, the genus *Cerastoderma* comprises two species: *C. glaucum* and *C. edule*. Rygg (1970) suggested that *C. glaucum *may have evolved from an ancestor of modern *C. edule *that got isolated when the Mediterranean basin was cut off from the Atlantic Ocean in the late Miocene. *C. edule* then evolved in the Atlantic, while *C. glaucum,* within the Mediterranean basin, acquired a wide tolerance range for salinity through selection in the context of varying salinity conditions during the Messinian Salinity Crisis (Brock, 1991). After the opening of the Strait of Gibraltar at the beginning of the Pliocene, *C. glaucum* spread northward in sheltered areas along the Atlantic coast (Brock, 1991). Already more than 30 years ago, it was widely discussed whether the Atlantic and the Mediterranean Sea populations of the “*Cerastoderma glaucum *complex” belonged to the same species, or whether an Atlantic species, *C. lamarcki, *should be distinguished. Currently, *C. lamarcki* is considered as synonymous to *C. glaucum*, but the subdivision into two species was suggested based on immunoelectrophoretical studies (Brock, 1987), karyotypes (Thiriot‐Quiévreux & Wolowicz, 1996), and chromosomal DNA differences (Brock & Christiansen, 1989). An allozyme variation study performed by Hummel, Wolowicz, and Bogaards (1994) also supported this division at a rank of subspecies. Genetic differentiation between *C. glaucum* populations has more recently been further studied based on more allozymes (Mariani, Ketmaier, & de Matthaeis, 2002; Nikula & Väinölä, 2003), mitochondrial DNA (Nikula & Väinölä, 2003; Tarnowska, Chenuil, Nikula, Féral, & Wolowicz, 2010), microsatellites (Tarnowska et al., 2010), and EPIC marker (Sromek et al., 2016). These studies did not support a subdivision of *C. glaucum* into distinct Mediterranean and the Atlantic–Baltic forms, as a major phylogeographic break was not found between Atlantic and Mediterranean populations, but inside the Mediterranean Sea (Nikula & Väinölä, 2003; Sromek et al., 2016; Tarnowska et al., 2010). The exact location of this newly discovered genetic break varied with the marker used (Figure 1). The deepest phylogeographic split in mtDNA grouped populations of the Aegean Sea and the Ponto‐Caspian region against the more western populations (Nikula & Väinölä, 2003; Tarnowska et al., 2010), whereas microsatellites rather indicated a separation of the Ionian Sea populations from the others (Tarnowska et al., 2010). The subsequent analysis of EPIC marker allele frequencies revealed the divergent character of both the Ionian Sea and Aegean–Ponto‐Caspian groups (Sromek et al., 2016). On the other hand, the relatedness of populations and the pattern of the range expansion into the Atlantic remained unresolved. Two different mitochondrial haplogroups with unexpected geographic disjunctions had been found in the Atlantic–Baltic region (Tarnowska et al., 2010). The first Atlantic haplogroup was found only outside of the Mediterranean and was shared between Ria Formosa (Portugal) and the Baltic Sea populations (haplogroup H1 in Figure 1). The second one was shared between the North Sea, Arcachon (French Atlantic coast), and Mediterranean Sea populations from Berre Lagoon and Sardinia (H2 in Figure 1). Two more haplogroups were described in Western Mediterranean: one shared between Sicily and Berre Lagoon populations (H3 in Figure 1), and another one unique for the Tunisia population (H4 in Figure 1). Multivariate analysis of EPIC and microsatellite allele frequencies suggested the existence of a cline in genetic differentiation from the Bay of Biscay to the Baltic Sea, but the Western Mediterranean populations appeared to be composed of a mixture of genotypes from different origins (Sromek et al., 2016). Such pattern of population admixture may indicate a complex population history (e.g., secondary contacts following allopatric divergence) or just biases introduced by the analysis of a small number of informative loci in the case of recent range expansion.

Accordingly, the existence of a strong genetic structure within *C. glaucum* has been acknowledged, but the discrepancy between markers and the low number of analyzed loci did not allow to conclude about a cessation of gene flow among intraspecific groups. In order to clarify this complex phylogeographic history through a RADseq approach, we genotyped 204 *C. glaucum* individuals from the same pan‐European panel of samples (from the Black Sea to the Baltic along the Mediterranean and Atlantic coasts) as in previous mitochondrial, microsatellite, and EPIC studies (Sromek et al., 2016; Tarnowska et al., 2010). This research was designed to analyze the genetic diversity of *C. glaucum* populations using an incomparably denser genome sampling, in order to (a) comprehensively reconstruct the phylogeographic relationships among populations, (b) describe divergence patterns across the genome, and (c) localize major genetic breaks and discuss their origin and maintenance.

## 2 MATERIALS AND METHODS

### 2.1 RADseq library preparation

In the present study, the same set of samples as used in previous studies by Tarnowska et al. (2010) and Sromek et al. (2016) was analyzed. DNA extracts from 500 individuals were visualized using agarose gel electrophoresis to determine the quality of DNA after 7 years of storage. The DNA quality and quantity differed substantially among individuals and populations, from highly degraded DNA to DNA with clear high molecular weight bands and concentration of around 70 ng/μl. Overall, 224 individuals of *Cerastoderma glaucum* from 14 previously analyzed samples (Figure 1) and 29 individuals from the sister species *Cerastoderma edule* (not analyzed before) from the Atlantic coast of Portugal, Sylt, and Danish fjords have been picked for RADseq analysis. Samples were selected based on DNA quality and quantity, in order to reach 10–17 individuals per population. DNA libraries were prepared from old DNA samples or new extractions from tissue stored in ethanol (noting that re‐extraction did not always give a better DNA yield). DNA extraction has been performed using GeneJET Genomic DNA Purification Kit (Thermo Scientific) following the manufacturer's instructions. The DNA concentration was measured using the Quant‐iT PicoGreen kit (Invitrogen). DNA samples were ranked according to concentration of double‐stranded DNA and divided into six quality groups (libraries) and normalized to the lowest concentration within each group. The RAD libraries were prepared following the protocol outlined in Etter, Bassham, Hohenlohe, Johnson, and Cresko (2011) with some modifications as described below. The gDNA was digested with the *Sbf*I high‐fidelity restriction enzyme (New England Biolabs). The predicted number of RAD loci for this enzyme was 15,435, assuming a 1.34 GB genome size (estimation from *Cerastoderma edule*; Rodríguez‐Juíz, Torrado, & Méndez, 1996) and a 40% GC content, based on radcounter computation (https://www.wiki.ed.ac.uk/display/RADSequencing). Ligation reactions were then prepared to attach 32 (two initial libraries) or 53 (four final libraries) different barcoded P1 adapters (IDT, Leuven, Belgium). The barcodes were 6 or 5 bp long and differed by at least two bases. To increase the amount of DNA in low‐concentration libraries, we adjusted the volume of digestion–ligation products for multiplexing from 20 μl per individual in the highest concentration library (beginning at a DNA concentration of 15 ng/μl) to 60 μl per individual in the lowest concentration library (DNA concentrations of around 2 ng/μl). After multiplexing, the samples were sheared in a Covaris sonicator for 75 s. Sonification efficiency and final quality of the libraries were checked on an Agilent Bioanalyzer. Final amplification in 16 PCR cycles was carried out using 1.5 µl of RAD amplification primers. All purification steps were performed using Macherey‐Nagel NucleoSpin Gel and PCR Clean‐up columns, except for the two last ones (before and after PCR reaction), for which the Agencourt AMPure XP beads have been used. Two initial libraries have been sequenced at Institut Pasteur de Lille, France, and the four final libraries at McGill University and Génome Québec Innovation Centre, Canada, on an Illumina HiSeq (100‐bp, single‐read format).

### 2.2 De novo RAD locus and SNP identification

Demultiplexing, filtering, and clustering of sequence reads were performed with pyrad v. 3.0.63 software pipeline (Eaton, 2014). Compared to the more frequently used stacks pipeline (Catchen, Amores, Hohenlohe, Cresko, & Postlethwait, 2011), pyrad, developed especially for phylogeographic and phylogenetic applications, allows for indel variation within and between samples, which is an advantage when considering genetically more‐distant samples (Eaton, 2014; Pante et al., 2015). Either no base mismatch in the sample‐specific barcode (for the four libraries with 53 adapters which differ at least 2 nucleotides) or one mismatch (for the two libraries with 32 adapters which differ at least 3 nucleotides) was allowed. The restriction site and barcode were trimmed from each sequence, bases with FASTQ quality score below 20 were replaced with *N*, and sequences having more than four undetermined sites were discarded. After demultiplexing, four individuals were excluded from further analysis, as one adapter probably failed to ligate, resulting in a very low number of reads in each library. Generally, the recommendations of Mastretta‐Yanes et al. (2015) were followed to explore and choose assembly parameter values that both increase the number of output loci and minimize the genetic dissimilarity between individuals from the same sampling location. Four similarity thresholds were tested: 0.8, 0.85, 0.9, and 0.94. The minimum number of samples displaying a given locus was set to 111 (more than half of *C. glaucum* individuals). The maximum proportion of individuals with shared polymorphic site in a locus was set to 50%. The remaining parameters were kept to default values. pyrad output was analyzed in the software r v. 3.3.1 (R Core Team, 2016). To compare datasets constructed with different thresholds, neighbor‐joining trees were constructed based on Euclidean distances among vectors of allele frequencies using the “ape” v. 3.5 package (Paradis, Claude, & Strimmer, 2004). For further analysis, only *C. glaucum* individuals having more than 50% of the mean number of loci per individual have been considered. Three individuals for which clustering did not coincide with geographic origins were excluded as potential labeling errors (one individual from the Aegean Sea which clustered with Ionian Sea populations, one individual from the Sylt population which clustered with Aegean–Black Sea populations, and one individual from the Berre Lagoon which clustered with Aegean–Black Sea populations). In a separate run of the pipeline with the same parameters, *C. edule* individuals were included and set as an outgroup. This setting allowed to input for *C. edule* individuals only the loci common to both species, and thus to root the phylogenetic tree.

### 2.3 Population genetic analyses

For population genetics analysis, pyrad vcf output files were converted into the Genepop format using pgdspider version 2.0.1.5 (Lischer & Excoffier, 2012), imported, and analyzed in the software R to further filter SNPs for analysis. Only biallelic SNPs with a minor allele frequency above 0.01 and genotyped in at least 80% of the individuals of each population were kept. The exact tests for Hardy–Weinberg equilibrium (HWE) with likelihood ratio as the test statistic were performed in “HWxtest” v. 1.1.8 R package (Engels, 2016). SNPs which failed the HWE test at *p* < 0.05 in more than two populations were excluded. Basic polymorphism descriptors (number of private SNP alleles per population, number of alleles per loci, observed and expected heterozygosity) were calculated in “poppr” v. 2.2.0 (Kamvar, Tabima, & Grünwald, 2014) and “adegenet” v. 2.0.1 R package (Jombart & Ahmed, 2011).

Pairwise *F*_ST_ values (Weir & Cockerham, 1984) were calculated in “StAMPP” v. 1.4 package (Pembleton, Cogan, & Forster, 2013) and visualized using nonmetric multidimensional scaling in “MASS” v. 7.3‐45 R package (Venables & Ripley, 2002). The confidence intervals were generated using 10,000 bootstrap replicates. A higher level of population structure (populations nested within geographic region) was tested using an AMOVA approach (Excoffier, Smouse, & Quattro, 1992) implemented in “poppr” R package. Significant deviation from random population structure was tested using the function randtest with 9,999 bootstrap replicates. Isolation by distance was tested by a Mantel test (Mantel, 1967) computed for pairwise comparisons of genetic and geographic distance matrices using “ade4” v. 1.7‐5 R package (Dray & Dufour, 2007). The *P*‐value of the regression factor was calculated using 9,999 bootstrap replicates. Geographic distance was measured by drawing a path along the shortest waterway between sampling sites using Google Earth.

Individual admixture coefficients were estimated from the genotypic matrix using the R function snmf from package “LEA” v. 1.4.0 (Frichot & François, 2015). For this analysis, we sampled one, randomly selected, SNP from each single RAD locus to avoid tight linkage among loci. Assuming *K* ancestral populations, the snmf function provides least‐squares estimates of ancestry proportions. This algorithm differs from the one implemented in structure (Pritchard, Stephens, & Donnelly, 2000), but the estimates of ancestry coefficients are similar for outcrossing species (Frichot, Mathieu, Trouillon, Bouchard, & François, 2014). To choose the optimal number of ancestral populations, this function estimates an entropy criterion that evaluates the quality of fit of the statistical model to the data using a cross‐validation technique. In addition, the appropriate number of genetic clusters was also identified using K‐means clustering as implemented in the “adegenet” package. To visualize relatedness between groups, a discriminant analysis of principal components (DAPC; Jombart, Devillard, & Balloux, 2010) was performed using clusters defined by K‐means clustering. The function xvalDapc was used to select the correct number of principal components for DAPC based on lowest root‐mean‐squared error criterion. Analyses of population genetic structure were performed on the whole dataset comprising all 14 populations and then separately on each main cluster to determine more precisely whether, within clusters, cryptic structure existed. To test whether filtering process for SNPs has not limited the ability to detect population admixture, we also repeated the structure analysis on dataset with less restrictive filtering (50% threshold for missing data and without HWE filtering).

To detect outlier SNP loci, a principal components analysis method was conducted using “pcadapt” v. 3.0.4 R package (Luu, Bazin, & Blum, 2016). This method does not require a priori population assignment and is specifically designed to detect outlier loci in the face of population structure (Duforet‐Frebourg, Luu, Laval, Bazin, & Blum, 2016). The optimal number of principal components (K) necessary to describe population structure was determined based on inspection of scree plot (Luu et al., 2016). The five principal components were retained for analysis. To detect outliers, we employed a false discovery rate (FDR) correction with “qvalue” v. 2.4.2 R package (Storey, Bass, Dabney, & Robinson, 2015) with a FDR cutoff of 10%.

To investigate unexpected pattern in mitochondrial DNA, associations between SNP alleles and Atlantic haplogroup I and haplogroup II were tested with Fisher's exact test in R. The *p*‐values for multiple comparisons were adjusted using the false discovery rate (FDR) method of Benjamini and Hochberg (1995). The dataset for this analysis included 48 individuals from Atlantic–Baltic region and 1,581 biallelic SNPs (not all SNPs were polymorphic within these individuals). Separately, the associations between SNP alleles and mitochondrial haplogroups were also investigated within Western Mediterranean cluster where divergent haplogroups were found in close geographic proximity. For this analysis, 33 individuals for which we had information about haplogroups were selected, and dataset with 1,788 biallelic SNPs was analyzed.

### 2.4 Phylogenetic analyses

To estimate the overall species tree, 24 individuals of *C. edule* which had more than 200 common loci with *C. glaucum* were included in analysis. Groups of *C. glaucum* identified by LEA structure analysis were used as a priori designated “species” in a coalescent analysis. The species tree was estimated using svdquartets (Chifman & Kubatko, 2014) as implemented in paup* v. 4.0a150 (Swofford, 1998). This method infers the relationships among quartets of taxa under a coalescent model. We used random quartet sampling from RAD locus sequences and nonparametric bootstrapping with 200 replicates to measure uncertainty in bipartitions.

## 3 RESULTS

An average of 2,790,052 reads per individuals were obtained. Mean coverage per locus was high for all tested similarity thresholds: 146 at 94%, 217 at 90%, and 222 at 85% and 80%. The number of consensus loci for each individual scaled with the sequence similarity threshold. Conservative clustering (i.e., 94% clustering vs. 80%) produced more loci per individual, but as a consequence, these loci were shared by less individuals (Figure S1). To assess whether true or erroneous loci were assembled, neighbor‐joining trees constructed on datasets with different similarity thresholds were visually compared (Figure S2). Individuals collected from the same population are expected to be genetically more similar in dataset with the smallest error rate (Mastretta‐Yanes et al., 2015). Based on this criterion, the similarity threshold of 80% was chosen for further analysis, as it not only maximized the amount of shared loci, but also minimized the genetic dissimilarity between individuals from the same sampling location, resulting in a diversity distribution that was the most biologically meaningful.

Clustering analyses resulted in a dataset containing 5,127 RAD loci for a total of 77,944 SNPs. The average number of SNPs per RAD locus was 15.2, ranging from 1 to 50 (Figure S3). A total of 71,505 (92%) SNPs were biallelic, 6,151 (8%) triallelic, and 288 (0.4%) tetra‐allelic. The entire RAD loci dataset was used for coalescent‐based species tree estimation. For population genetic analysis, we further excluded 68,876 SNPs with more than 20% of missing data per population; 5,536 SNPs with minor allele frequency below 1%; and 405 nonbiallelic SNPs and 165 SNPs which failed the HWE test in more than two populations. These filtering resulted in a dataset of 2,962 SNPs from 658 RAD loci.

### 3.1 Genetic diversity within and among *C. glaucum* populations

When considering the geographic distribution of the genetic diversity, it is clear that Mediterranean and Black Sea locations demonstrate higher values of expected heterozygosity as compared to the Atlantic, North Sea, and Baltic populations (pairwise Wilcoxon signed‐rank test, *p* < 0.0001). The mean number of alleles per locus and the number of private alleles per population also decreased from the Black Sea and Eastern Mediterranean to the Baltic Sea (Table 1).

**Table 1:** Genetic diversity within *Cerastoderma glaucum* populations calculated from data of 2,962 biallelic SNP markers

Geographic location	Site code	Region	*N*	Private	A	*H*_obs_	*H*_exp_
Tvärminne, Finland	FI	Baltic Sea	15	2	1.2836	0.0681	0.0778
Gulf of Gdansk, Poland	GD		19	2	1.3278	0.0763	0.0897
Sylt, Germany	AL	North Sea	13	4	1.2964	0.0734	0.0876
Lake Veere, Netherlands	LV		10	3	1.2741	0.0801	0.0831
Arcachon Bay, France	AR	Atlantic Ocean	16	9	1.3106	0.0763	0.0859
Ria Formosa, Portugal	PT		14	7	1.3805	0.0788	0.0985
Berre Lagoon, France	BL	Western Mediterranean	15	10	1.4841	0.1069	0.1283
Sardinia, Italy	SA		11	7	1.3940	0.0874	0.1152
Tunis Bay, Tunisia	TU		16	9	1.4274	0.0762	0.1179
Sicily, Italy	SI		16	30	1.5240	0.0960	0.1371
Gialova Lagoon, Greece	GI	Ionian Sea	15	39	1.4105	0.1023	0.1187
Etolikon, Greece	ET		16	22	1.4747	0.1090	0.1239
Porto Lagos, Greece	GR	Aegean–Black Sea region	14	41	1.4851	0.0957	0.1286
Constanta, Romania	RO		14	43	1.4605	0.0846	0.1217

Note

*N*, number of individuals included in the final analyses; Private, the number of private alleles for each population; *A*, average number of alleles per locus; *H*_obs_, the average observed heterozygosity per locus; *H*_exp_, the average expected heterozygosity per locus.

**Figure 2:**
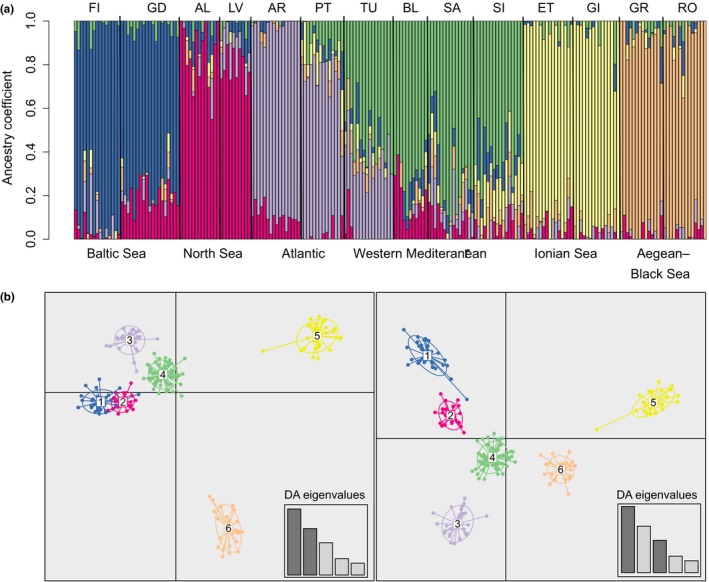
Estimation of population genetic structure of *Cerastoderma glaucum*. Plots of posterior probabilities of group assignment of each individual based on admixture analysis (a). The color proportion for each bar represents the posterior probability of assignment of each individual to one of six clusters of genetic similarity. Subdivision of the six *C. glaucum* clusters according to the DAPC (b). Scatter plot of the first three components of the discriminant analysis (DA). Dots represent individuals; 95% inertia ellipses are included for each cluster

**Figure 3:**
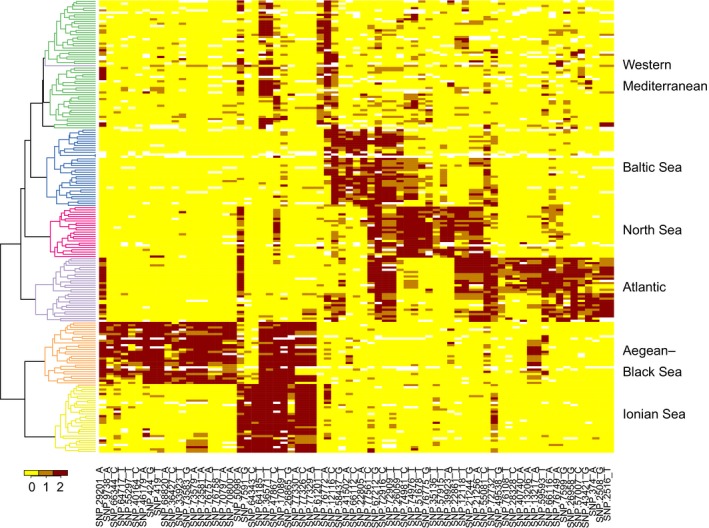
Minor allele frequencies at outlier loci. Colors indicate the number of minor SNP alleles for each *Cerastoderma glaucum* individual at 71 outlier loci. Minor allele was defined based on the pool of all populations. The dendrogram shows the results of a hierarchical clustering based on Euclidean distances among vectors of allele frequencies

Population structure was strong (Tables S1, S2, Figure S4); mean pairwise *F*_ST_ ranged from 0.030 (Porto Lagos vs. Constanta) to 0.434 (Tvärminne vs. Gialova Lagoon). In the population structure analysis, a model with *K* = 6 was found to fit best to the data, as both cross‐entropy criterion (from “LEA” package) and Bayesian information criterion (from “adegenet” package) converged on this number of suggested clusters (Figure S5). Admixture coefficient bar plots clearly showed these six groups: Baltic, North Sea, Atlantic, Western Mediterranean, Ionian Sea, and Aegean–Black Sea (Figure 2a). Finer substructure could be detected when individual regions were analyzed separately (Figure S6). Within the Baltic, North Sea, Atlantic, Western Mediterranean, and Ionian Sea groups, components corresponding to sampling sites could be distinguished. In contrast, a focused analysis within the Aegean–Black Sea group did not support the existence of subgroups. All six groups derived by structure analysis were also well discriminated by DAPC (Figure 2b). The structure analysis based on a bigger dataset and less restrictive SNPs filtering (50% threshold for missing data, without HWE filtering) gave very similar results (Figure S7). The analysis of molecular variance (AMOVA) revealed a significant population structure among regions (*p* = 0.0001). The major part (22%) of the genetic variation that was not attributable to variation within individuals (which amounted to 57% of total variance) was partitioned across geographic regions, whereas only 8% of the variation was accounted for by differences among populations nested within the geographic regions (Table S3). A total of 71 outliers were detected out of the 2,962 biallelic SNPs. These highly differentiated SNPs came from 54 RAD loci and strongly differentiated the six regional *C. glaucum* clusters (Figure 3).

**Figure 4:**
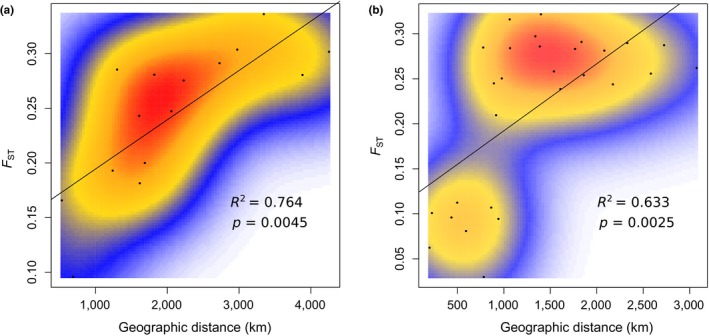
Plots of the genetic distance (*F*_ST_) between *Cerastoderma glaucum *populations against their geographic distance: Mediterranean and Black Sea populations (a), Atlantic populations, from Portugal coast to the Baltic Sea (b). Local density of points was measured using a two‐dimensional kernel density estimation

**Figure 5:**
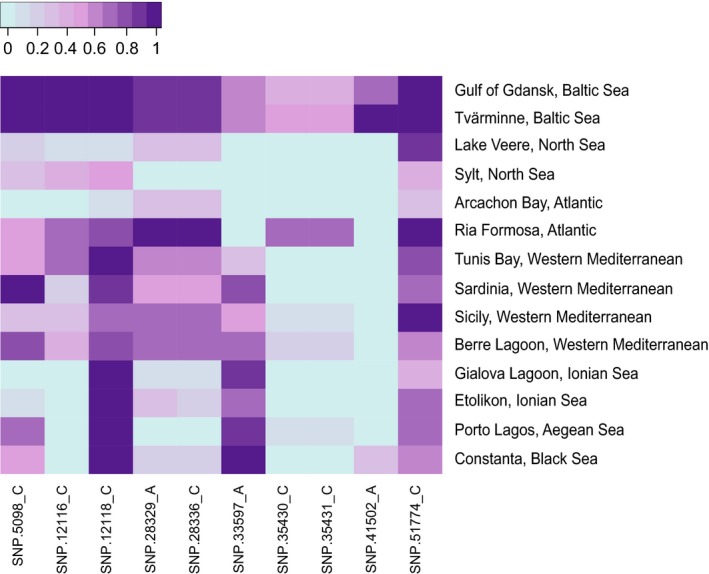
Allele frequencies at the 10 SNPs most strongly associated with mitochondrial haplogroup I and haplogroup II in Atlantic populations of *Cerastoderma glaucum*. See Figure 1 for geographic distribution of mtDNA haplogroups

The Mantel test showed a significant correlation of genetic differentiation with geographic distance (*p* = 0.0001), and the coefficient of determination *R* ^2^ equaled 0.686. Geographic distance explains more genetic variance in Atlantic populations (from Portugal to Baltic Sea) as compared to Mediterranean Sea populations (Figure 4). As much as 128 SNPs were found to be associated with two Atlantic mitochondrial haplogroups (FDR‐corrected *p* < 0.05) from Tarnowska et al. (2010). The spatial distribution of 10 SNPs with the lowest *p*‐value is shown in Figure 5. Further, frequencies of 18 SNPs were unevenly distributed between the three Western Mediterranean haplogroups.

### 3.2 Phylogenetic inferences

**Figure 6:**
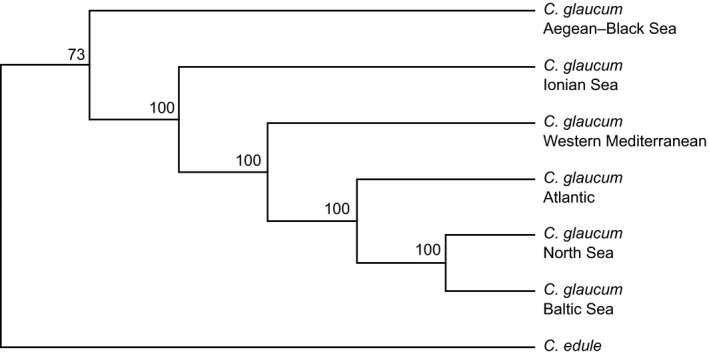
Species tree built from the genetic clusters of *Cerastoderma glaucum,* as resolved by quartet sampling using svdquartets. Numbers correspond to bootstrap replicates that support the respective split. Branch lengths are arbitrary (topology tree)

On the coalescent‐based species tree, the Aegean–Black Sea cluster of *C. glaucum *was located nearest to the root of the tree, outward from which were branches that corresponded, sequentially, to the clusters from Ionian Sea, Western Mediterranean, Atlantic, North Sea, and Baltic (Figure 6). All relationships, except the placement of *C. edule*, received high uniform support (bootstrap values of 100%).

## 4 DISCUSSION

### 4.1 Unprecedented resolution for phylogeography of *C. glaucum*

The mean pairwise *F*_ST_ value of 0.27 estimated from SNPs (Table S1 and S2) was considerably higher than the one estimated previously from microsatellite loci for the same set of populations (0.16; Tarnowska et al., 2010). This lower value was mainly due to an underestimation of the differentiation between the divergent Aegean–Black Sea and other populations. Values of mean pairwise *F*_ST_ between Aegean–Black Sea populations and populations from other regions were 2–3 times higher from SNPs compared with estimates from microsatellites. In contrast, the mean pairwise *F*_ST_ value estimated from SNPs between Baltic and North Sea populations was only slightly higher than the one estimated from microsatellites (0.22 vs. 0.18, respectively). These observations are in accordance with the hypothesis that homoplasy is likely to affect microsatellites when populations are distantly related (Selkoe & Toonen, 2006). The clustering analysis clearly identified six groups of *C. glaucum* populations, which corresponded to geographic regions. These results differ from previous studies on the same samples (Sromek et al., 2016; Tarnowska et al., 2010) mainly in resolution. Strong genetic structuring of *C. glaucum* populations was revealed across all three studied marker types (mtDNA, microsatellites, and EPIC marker), but the location of major phylogeographic breaks varied with the marker used (Figure 1) and the number of genetic clusters was ambiguous (Sromek et al., 2016; Tarnowska et al., 2010). Six regional clusters were much more clearly separated with RADseq data than was possible based on traditional genetic markers. Mitochondrial DNA analysis suggested that the divergent Aegean–Black Sea lineage of *C. glaucum* diverged first, but the relatedness among more western groups could not be solved (Nikula & Väinölä, 2003). The coalescent‐based species tree (Figure 6) estimated from the genetic clusters of *C. glaucum* revealed a phylogeny that largely agrees with the plausible postglacial colonization route of this species from the Eastern Mediterranean to the Baltic Sea. The Baltic and North Sea cluster shares a more recent common ancestor with the Atlantic cluster than it does with the Western Mediterranean. The RADseq approach, by providing a much denser genome‐wide sample of genetic diversity, overwhelms sampling error and provides a precise estimate of phylogeographic relationships.

For species with low dispersal ability, a higher genetic similarity is expected between neighboring populations than between distant ones (Wright, 1943). This pattern is very pronounced in *C. glaucum*—all populations were significantly differentiated and spatially structured according to an isolation‐by‐distance pattern. Nevertheless, the identification of significant IBD pattern does not necessarily imply spatially homogeneous gene flow and the absence of barriers to gene flow (Aurelle et al., 2011; Garnier, Alibert, Audiot, Prieur, & Rasplus, 2004). The pattern of IBD can differ from one region to the other and depends on the spatial scale considered. For instance, in the stepping‐stone model (Kimura & Weiss, 1964), the demes may constitute elementary units which can be recovered by clustering analysis (Aurelle & Ledoux, 2013). IBD patterns found in C*. glaucum* were quite different when considering Atlantic (from Portugal to Baltic Sea) and Mediterranean populations separately (Figure 4). The geographic distance explained a greater part of the genetic differentiation in Atlantic than in Mediterranean Sea. In the Mediterranean Sea, two patches of distant and differentiated populations have been observed, in contrast to the continuous cline of genetic differentiation detected from the Portugal coast to the Baltic Sea. The Ionian Sea and Aegean–Black Sea clusters were the most differentiated from the rest, as determined by DAPC (Figure 2b) and pairwise *F*_ST _(Figure S4). Despite the close geographic proximity of the Ionian and Aegean basins, these two clusters were strongly differentiated with the smallest proportion of admixture, which suggests the existence of efficient and long‐lasting barriers to gene flow in the Eastern Mediterranean. Outside of the Eastern Mediterranean, genetic clusters seemed to be more linked to the effect of limited dispersal than to the barriers to gene flow, as no sharp genetic discontinuity was observed across well‐known marine biogeographic boundaries, for example, between Mediterranean and Atlantic basins (Patarnello, Volckaert, & Castilho, 2007). Individuals from the Baltic, North Sea, Atlantic, and Western Mediterranean populations were inferred to belong mostly to their corresponding clusters, but admixture from neighboring region was clear (Figure 2a). The pattern of spatial autocorrelation is problematic for clustering analyses and can lead to the inference of spurious barriers to gene flow (Aurelle & Ledoux, 2013; Meirmans, 2012). Outside of the Eastern Mediterranean, clusters of *C glaucum* populations can theoretically reflect sampling patterns rather than intrinsic genetic structure. However, AMOVA suggested that the average genomic divergence tends to be lower within than between these groups, as would be expected if these clusters were indeed meaningful groups that reflect evolutionary relationships. An alternative grouping, which pooled Baltic and North Sea populations together (as the most recently diverged), explained a slightly lower proportion of variance (22% vs. 23%). Thus, there must exist a significant proportion of SNPs that distinguish even the youngest split, between the Baltic and North Sea clusters. Furthermore, investigation of allele frequencies at the most differentiated loci (Figure 3) revealed that gradients of allele frequencies could be observed in some loci, but also that abrupt genetic discontinuities nevertheless occurred between all *C. glaucum* regional clusters. Therefore, overall genetic structure of *C. glaucum* is characterized by the pattern of IBD, but gene flow between regions seems to be limited in substantial parts of the genome.

Comparisons of IBD patterns within and between clusters would be helpful to further distinguish between barriers to gene flow and limited dispersal. Unfortunately, our data do not allow for such a test. Only two populations were sampled within Baltic, North Sea, and Atlantic regions. Furthermore, all population pairs within these regions are separated by smaller geographic distance than population pairs between regions. The only exception is the Arcachon Bay population which is separated by a distance of ~1,600 km from the Ria Formosa population but is only ~1,300 km away from the Lake Veere population. In this case, genetic differentiation for within‐clusters comparison (*F*_ST_ = 0.18, Arcachon Bay/Ria Formosa) is indeed smaller than for between‐clusters comparison (*F*_ST_ = 0.28, Arcachon/Lake Veere), despite a larger geographic distance. Within the Western Mediterranean cluster, genetic differentiation was not correlated with geographic distance (*p* = 0.542, *R* ^2^ = −0.063, data not shown), but the number of pairwise comparisons among four populations was probably too small to provide a reliable estimate of correlation. Interestingly, a finer geographic structure was visible within most clusters, but not within the Aegean–Black Sea clusters (Figure S6). The Black Sea population is separated from the Aegean Sea population not only by a distance of about 784 km, but also by the Turkish Strait System. Migration between Mediterranean and Black Sea is considered to be essentially one way for pelagic larvae due to the surface currents flowing to the Aegean Sea, while the opposite is prevented by the sinking of the more saline Mediterranean waters into deep anoxic waters of Black Sea (Kalkan, Karhan, Bilgin, & Hemond, 2016). The pairwise *F*_ST _value of 0.03 between Porto Lagos (Aegean Sea) and Constanta (Black Sea) populations was nevertheless the lowest one in the present dataset (Table S1). Population pairs within clusters that were separated by similar or smaller geographic distances (e.g., Sylt/Lake Veere, Berre Lagoon/Tunis Bay, Gulf of Gdansk/Tvärminne) were more differentiated. A recent introduction of Aegean Sea populations into the Black Sea is not likely, as *C. glaucum* is known to be present in the Black Sea region through a 400‐ to 500‐ky‐old fossil record (Zubakov, 1988). On the other hand, there is a possibility that *C. glaucum* disappeared from the Black Sea due to the salinity drop during the last glacial period and reentered when Mediterranean waters flooded to the Black Sea about 7,500 years ago (Nikula & Väinölä, 2003). In this case, a low genetic divergence may be explained by the short period of time that has passed since the species recolonized the Black Sea, not long enough to allow differentiation to take place. For instance, probably due to large effective population size, two populations of mussels have been shown to be demographically independent for thousands of years while not departing from genetic panmixia (Fraïsse, Belkhir, Welch, & Bierne, 2016). Whatever the scenario, the phylogeographic pattern of *C. glaucum* in this region is quite unusual. Studies of genetic differentiation between the Mediterranean and Black Sea reported a decrease in genetic diversity from the Mediterranean Sea to the Black Sea (Patarnello et al., 2007). Both Aegean and Black Sea populations of *C. glaucum* have high numbers of private alleles, and such a decrease in diversity was not observed in our data (Table 1). The high genetic diversity of both Aegean and Black Sea populations suggests that potential recolonization was not accompanied by repeated founder effects, contrary to the pattern observed in the Atlantic, where a strong decline in genetic diversity is observed. It is therefore possible that connectivity patterns and/or population dynamics differ substantially between regions. The populations of *C. galucum* from North Sea and Baltic have one summer spawning period, whereas the southern populations from Western Mediterranean spawn few times during the year (Machreki‐Ajmi, Rebai, & Hamza‐Chaffai, 2013; Tarnowska, Wolowicz, Chenuil, & Feral, 2009). Thus, more frequent larval releases together with other factors, for example, plasticity in larval life duration related to environmental conditions, larval behaviors, or vertical position of larvae within the water column (Becker, Levin, Fodrie, & McMillan, 2007), may account for this variable connectivity pattern among regions.

### 4.2 Postglacial range expansion and mitochondrial DNA surfing

The population structure within *C. glaucum* was found to be consistent with a pattern of genetic diversity loss along a likely postglacial colonization route from the Mediterranean region to the Baltic Sea. In comparison with Mediterranean populations, populations from Atlantic, North Sea, and Baltic demonstrated markedly reduced levels of genetic diversity (Table 1). These observations support that the Atlantic range of *C. glaucum* was colonized or recolonized relatively recently, after the last glacial maximum. During the last glacial maximum (18,000–10,000 years ago), the southern ice limit in Europe was probably located at 43°N (northern Spain) (Frenzel, Pécsi, & Velichko, 1992); thus, suitable habitats for coastal organisms were restricted to the Portugal coast and southward.

It has been shown that new mutations arising at the front of a range expansion can occasionally travel with the wave of advance, be carried over long distances, and reach very high frequencies in the newly colonized areas (Excoffier & Ray, 2008; Klopfstein, Currat, & Excoffier, 2006). The probability of this phenomenon called allelic surfing increases when local deme size is small, when migration rate is low, and when populations at the edge of expansion grow rapidly (Klopfstein et al., 2006; Travis et al., 2007). These theoretical expectations fit to the *C. glaucum* range expansion model along Atlantic coast and can nicely explain the unexpected geographic disjunction found in the mtDNA analysis (Tarnowska et al., 2010). The haplotypes from the Ria Formosa population (Atlantic coast) were closely related, but not identical, to haplotypes from Baltic Sea populations (haplogroup H1 in Figure 1). Moreover, the haplotypes from North Sea and Arcachon Bay populations resembled those from Berre Lagoon and Sardinia, both Mediterranean populations (H2 in Figure 1). The scenario of the mitochondrial DNA surfing requires the presence of both Atlantic mtDNA haplogroups in ancestral populations prior to the northern expansion. During the Atlantic colonization, haplogroup H2 spread over the whole area, while haplogroup H1 was transferred on the wave of expansion to the Baltic Sea, where it reached fixation. This hypothesis further assumes the subsequent almost complete extinction of haplogroup H1 everywhere in the Atlantic except in the Baltic Sea and Ria Formosa populations (one individual with this haplogroup was found also in Lake Veere, North Sea). Taking into account the unstable population dynamic in lagoon habitats, a complete loss of one of the two haplogroups due to stochastic sampling is likely. The strong genetic drift in small, isolated populations and the frequent bottlenecks probably contributed to further, postcolonization reduction in genetic diversity. In the Baltic Sea, *C. glaucum* forms continuous populations along a large part of the coastal zone, but not in the North Sea nor along the Atlantic coast, where tidal amplitudes are high, and suitable habitats are thus scarce (Boyden & Russell, 1972; Brock, 1979). The Atlantic and North Sea populations of *C. glaucum* are moreover marked by temporal variations in population size and frequent extinctions (Labourg & Lasserre, 1980; Reise, 2003). Alternatively, haplogroup H1 can occur in very low frequencies in Arcachon Bay and North Sea populations, but was not sampled in previous mitochondrial studies. This strong spatial sorting of haplogroups could also be further maintained by mito‐nuclear coevolution (Burton & Barreto, 2012; Hill, 2016).

Mitochondrial DNA can be more prone to allele surfing than nuclear DNA because of its lower effective population size. However, if the scenario of mitochondrial DNA surfing on the wave of expansion is true, we also should find some nuclear alleles which behaved in a similar way. The spatial distribution of successfully “surfed” alleles should form clines with frequency increasing in the direction of the range expansion (Travis et al., 2007). Analysis of allele frequencies among populations in the most differentiated SNPs revealed many of such clines with low frequency in Western Mediterranean and high frequency in populations from the Atlantic–Baltic sector (Figure 3). As it is expected under the allele surfing scenario, some of these alleles attained very high frequencies or even fixation in peripheral Baltic Sea populations. Interestingly, allele frequencies for SNP_28329 and SNP_28336, with high values in Portugal and both Baltic Sea populations, mimicked the spatial distribution of haplotype H1 in Atlantic (Figure 5). Thus, a great part of genetic variation in *C. glaucum* seems to be spatially segregated because of the stochastic processes associated with a recent colonization of the Atlantic coast, and this can explain the poor resolution and discordant patterns found in previous studies. Mitochondrial surfing is rarely reported in the literature, but our scenario is similar to the one described by Streicher et al. (2016) for the Texas coral snake *Micrurus tener*. As noticed by the authors, circumstances under which mitochondrial surfing can be detected are rare: Divergent haplotypes need to be maintained in sympatry before range expansion, and the dispersal capability of species has to be limited.

In species with high dispersal ability, population differentiation that can arise during a range expansion is expected to be transient (Excoffier, Foll, & Petit, 2009). The low dispersal ability of *C. glaucum* together with its fragmented habitat could lead to a longer persistence of this structure. However, a clear‐cut separation of mitochondrial haplogroups was also observed among geographically close populations in Western Mediterranean (Tarnowska et al., 2010). The haplogroup H3 was found to be shared between Sicily and Berre Lagoon populations, while the divergent haplogroup H4 was found only in Tunis Bay (Figure 1). Such patchy distribution of haplogroups may have arisen by stochastic loss of lineages during initial colonization of lagoons, but it is intriguing why it is maintained in the face of nuclear gene flow. In our analysis, Western Mediterranean populations were separated by similar genetic distance, regardless of whether they shared the same haplogroup or not (Table S1, Figure S4). Such strong spatial sorting of mitochondrial haplogroups in the face of nuclear gene flow can suggest that some form of selection might be involved. This hypothesis is supported by our identification of nonrandom mito‐nuclear associations that can act as semipermeable barriers to gene flow and prevent mitochondrial DNA introgression (Burton & Barreto, 2012; Hill, 2016). In addition, environmental variation can drive a selection for different, locally adapted sets of mtDNA and nuclear‐encoded genes with mitochondrial functions (N‐mt genes). The fitness effects of these sets could depend on local environmental condition, for example, temperature or dissolved oxygen (Ballard & Whitlock, 2004; Hill, 2016). The involvement of exogenous or endogenous selection can help to explain the present‐day maintenance of a patchy distribution of the mitochondrial haplogroups, but to formally test this hypothesis, more data would be needed.

### 4.3 Heterogenous genomic differentiation

Several mechanisms may cause genetic differentiation between marine species populations such as vicariance processes, caused by historical barriers, oceanographic discontinuities, local adaptation, and limited dispersal capabilities (Palumbi, 1994). The spatially isolated nature of costal lagoons, at both present and past sea levels, may lead to significant population differentiation in allopatry, as highlighted by the studies of the Mediterranean goby *Pomatoschistus tortonesei* (Mejri et al., 2012), the pipefish *Syngnathus abaster* (Sanna et al., 2013), and the lagoonal sand smelt *Atherina lagunae* (Trabelsi et al., 2004). Previous studies of lagoon species were based mainly on few genetic markers, but our genome‐wide SNP analysis indicated that genetic differentiation between geographically isolated populations can be widespread across the genome and that many of the loci exhibit concordant genetic structure. Current equilibrium between migration and genetic drift seems to be mainly responsible for the observed strong genetic population structure. On the other hand, oceanographic discontinuities and geographic distance alone are not sufficient to explain the observed patterns in *C glaucum*, because genomic differentiation was found to be highly heterogeneous across the genome.

An elevated level of genetic differentiation at specific loci is commonly interpreted as a consequence of divergent selection (e.g., Nosil et al., 2009). However, the proportion of outlier loci (~2.4% of the studied markers) detected in *C. glaucum* is too high to be easily explained solely by local adaptation (Bierne, Welch, Loire, Bonhomme, & David, 2011). The majority of the outliers in the Atlantic–Baltic sector probably come from changes in allele frequencies associated with range expansion. The gradients of allele frequencies can be generated by recurrent founder events followed by low migration and allele surfing (Excoffier et al., 2009). On the other hand, gradients of allele frequency determined by environmental variation or resulting from a selective sweep can look very similar (Excoffier et al., 2009). Our results do not allow to discriminate between the effects of past demography and selection, but it is very likely that several spatially variable selective processes may act on a number of phenotypic traits among populations. Along the Atlantic coast, strong ecological gradients occur and substantial differences in cockle morphometry, biochemical composition of the cockle tissues, and in physiological parameters have been detected among populations of *C. glaucum* from Northern Europe, the French Atlantic coast, and the Western Mediterranean (Brock & Wolowicz, 1994; Tarnowska et al., 2009). Some of these physiological differences may as well reflect phenotypic plasticity, but could also, at least for some of them, indicate genetically based adaptations. Hence, it is likely that allele frequencies at outlier loci among Atlantic–Baltic clusters do represent the joint result of past demographic history and selection acting at multiple loci.

Contrary to the Atlantic–Baltic sector, there is no clear association between ecological variables and allele frequency shifts at outlier loci in the Mediterranean Sea. Strong genetic divergence at outlier loci between the Ionian Sea and Aegean–Black Sea clusters is at odds with the close geographic proximity of Aegean and Ionian Sea basin. Furthermore, the lagoon‐like character of *C. glaucum* habitat means that the environmental conditions it experiences during the year can be similarly extreme in the entire Mediterranean Sea. This species is characterized as being highly euryhaline and eurythermic (Ansell, Barnett, Bodoy, & Massé, 1981; Brock, 1991). In the Eastern Mediterranean, it lives in lagoons where salinity varies throughout the year from 13 PSU in spring up to 60 PSU in autumn (Koutsoubas, Arvanitidis, Dounas, & Drummond, 2000). Therefore, the high proportion of outlier loci found between Mediterranean clusters of *C. glaucum* populations is more likely the consequence of endogenous genetic barriers, created by genetic incompatibilities than a product of local adaptation. The endogenous genetic barriers are likely to impede neutral gene flow in a substantial proportion of genome (Barton & Hewitt, 1985; Wu, 2001) and tend to coincide with physical barriers to gene flow (Bierne et al., 2011). Recent studies demonstrated that genetic incompatibilities (i.e., combination of alleles involved in negative epistatic interactions) can be widespread even within species (Corbett‐Detig, Zhou, Clark, Hartl, & Ayroles, 2013; Ono, Gerstein, & Otto, 2017). Alleles that work well together within a given genetic group can perform poorly when combined with alleles from other groups, thus substantially reducing the fitness of hybrid individuals (Cutter, 2012). Endogenous selection on these genetic incompatibilities can significantly hamper gene flow following secondary contact, ultimately leading to postzygotic reproductive isolation (Barton & de Cara, 2009; Wu, 2001). The existence of a secondary contact zone within the Eastern Mediterranean is very likely, because of the close geographic proximity of three divergent genetic groups (Western Mediterranean, Ionian Sea, and Aegean–Black Sea clusters). The analysis based on all SNPs revealed that the Ionian Sea and Aegean–Black Sea populations have the highest numbers of private alleles and are the most differentiated from the rest. Thus, these groups were effectively isolated for a long time and could adapt to similar selection pressures via different genetic changes, accumulating genetic incompatibilities. In this context, outlier loci identified between these groups can be candidate loci that can be investigated regarding their contribution to the postzygotic isolation mechanisms and speciation.

It should be noted that there have been a variety of hypotheses concerning taxonomical divisions within *C. glaucum*. The cockles present a high variability in terms of morphology, which has led some authors to propose numerous species and subspecies which are currently treated as synonyms of *C. glaucum *or *C. edule* (Grossu, 1962; Mars, 1951). The subdivision into an Atlantic *Cerastoderma lamarcki* and a Mediterranean *C. glaucum* had been suggested based on immunoelectrophoretical, karyotype, and chromosomal DNA differences (Brock, 1987; Brock & Christiansen, 1989; Thiriot‐Quiévreux & Wolowicz, 1996). More recent studies based on a limited number of genetic markers did not support the subdivision between Mediterranean and Atlantic forms of *C. glaucum*, as a major phylogeographic break had been found in the Eastern Mediterranean (Mariani et al., 2002; Nikula & Väinölä, 2003; Sromek et al., 2016; Tarnowska et al., 2010). Our genome‐wide analysis confirmed the split between Western and two Eastern Mediterranean lineages, but also indicated that the Atlantic, North Sea, and Baltic populations of *C. glaucum* also constitute isolated entities. Thus, the “*Cerastoderma glaucum *complex” appears as a particularly intricate example, illustrating both the sampling variation biases introduced by the analysis of a limited number of loci and the important evolutionary role of fragmented lagoon habitats.

## CONFLICT OF INTEREST

None declared.

## AUTHOR CONTRIBUTIONS

M.W., D.F., R.L., and P.F. contributed to the ideas and project design. L.S. and D.F. performed the experiments and analyzed the data. L.S. led the manuscript writing.

## Data Availability

VCF file of individual SNP genotypes and aligned RAD sequences used for svdquartets analysis are available on the Dryad Digital Repository: https://doi.org/10.5061/dryad.c3770vq.
