# Supplementary figures and images for: Next‐generation phylogeography of the cockle Cerastoderma glaucum: Highly heterogeneous genetic differentiation in a lagoon species
Source: Ecol Evol. 2019 Mar 27;9(8):4667–82. doi: 10.1002/ece3.5070 (PMC6476780; doi:10.1002/ece3.5070)

A.

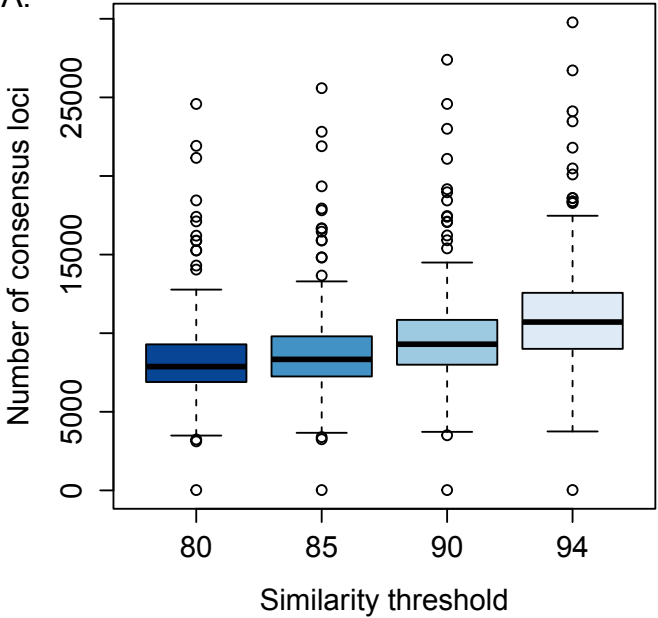

B.

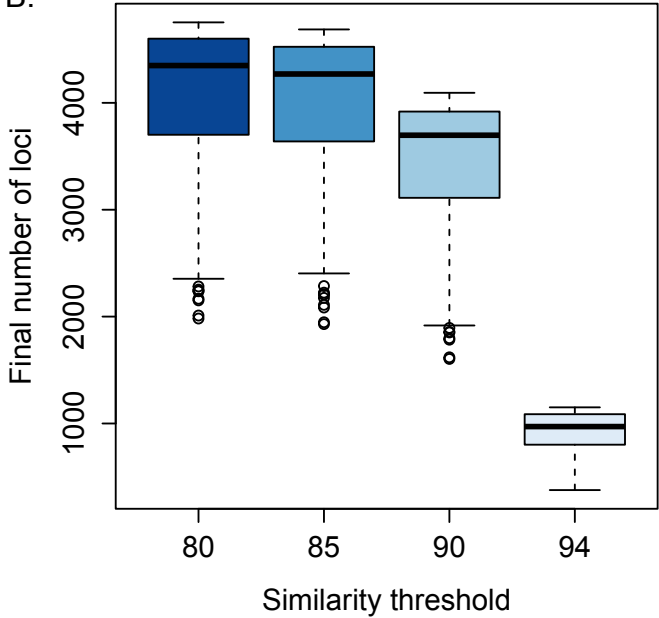

Supplement: Supplementary file 1 [file ECE3-9-4667-s001.pdf]

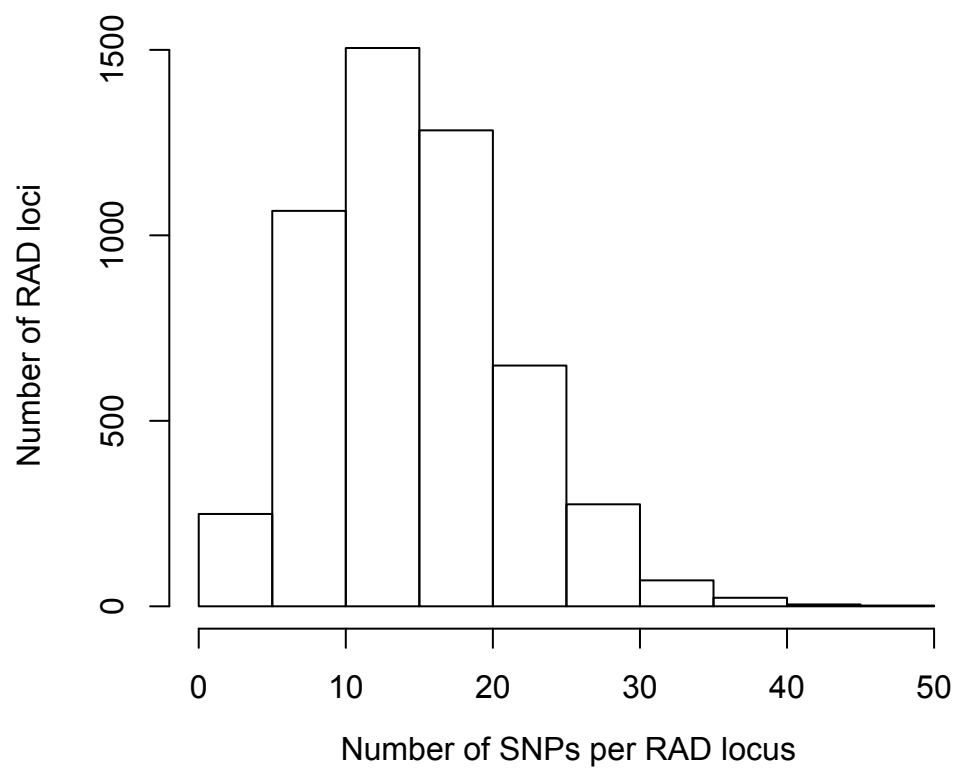

Supplement: Supplementary file 3 [file ECE3-9-4667-s003.pdf]

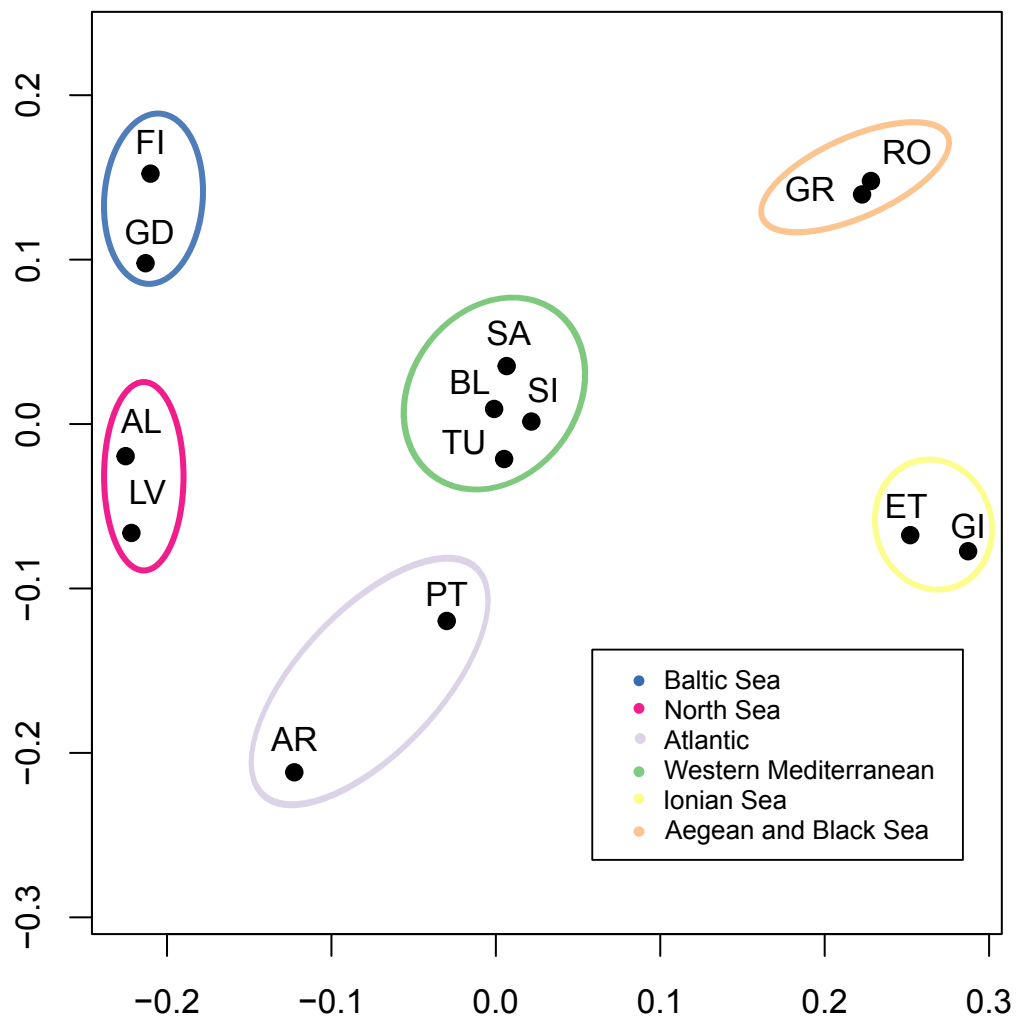

Supplement: Supplementary file 4 [file ECE3-9-4667-s004.pdf]

A.

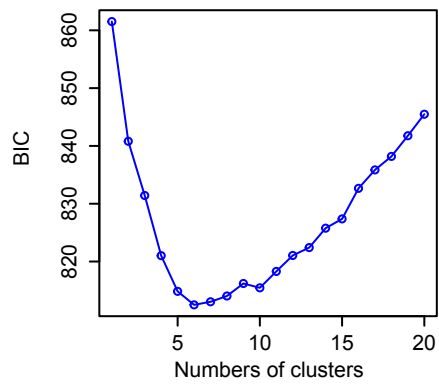

B.

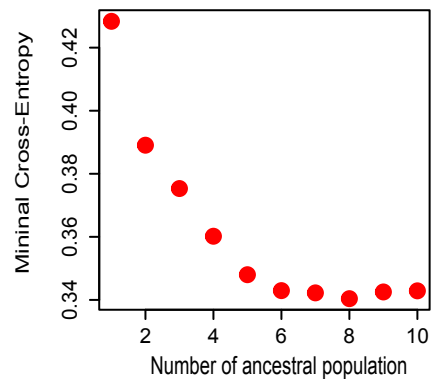

Supplement: Supplementary file 5 [file ECE3-9-4667-s005.pdf]

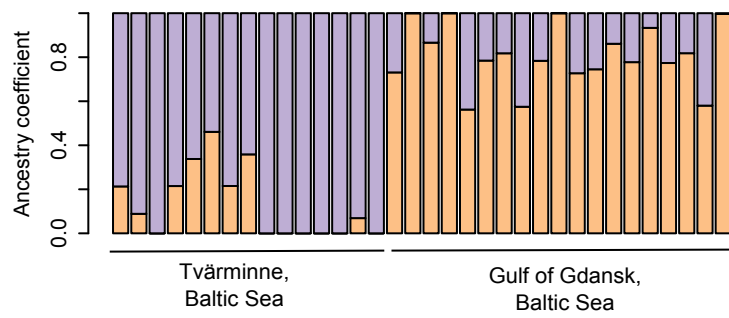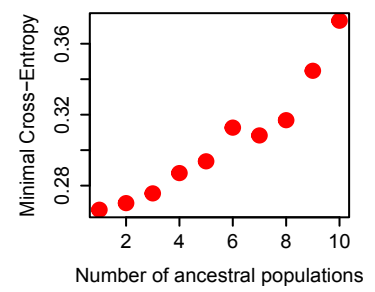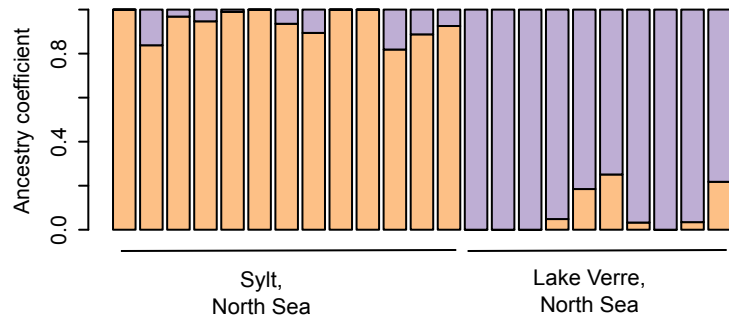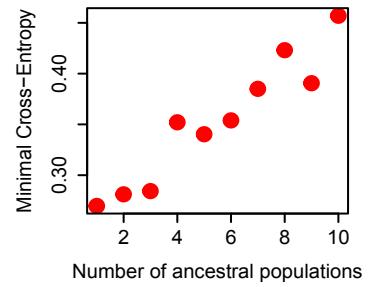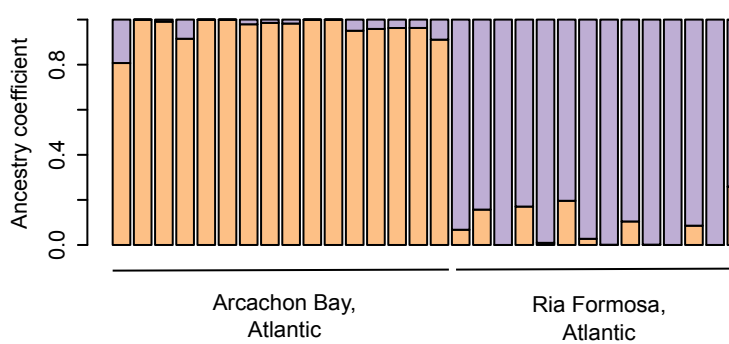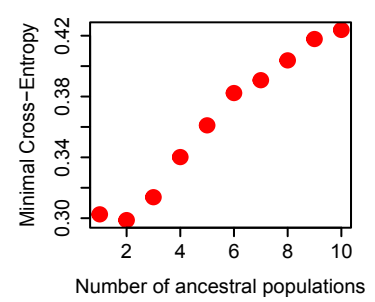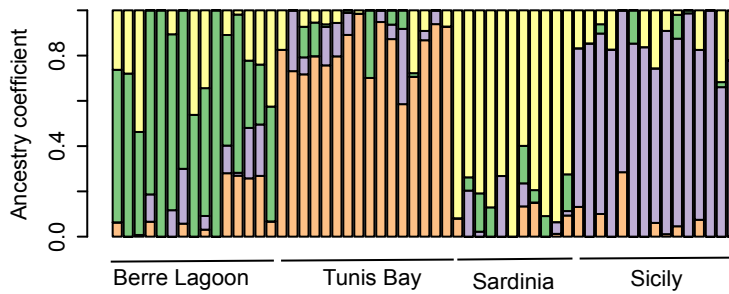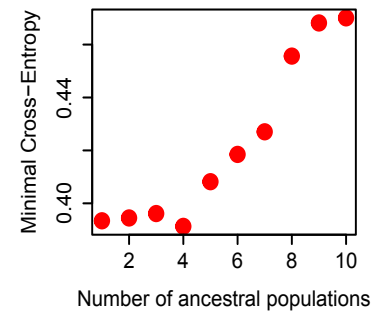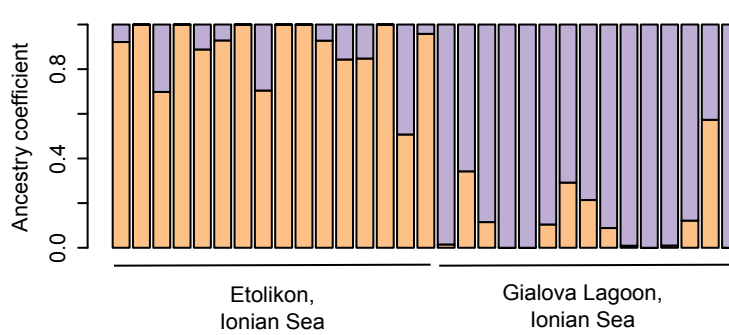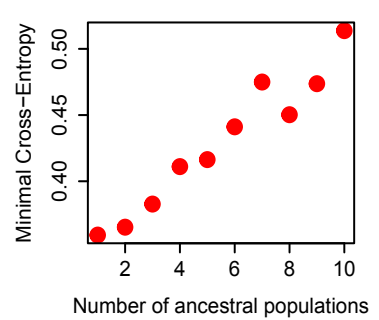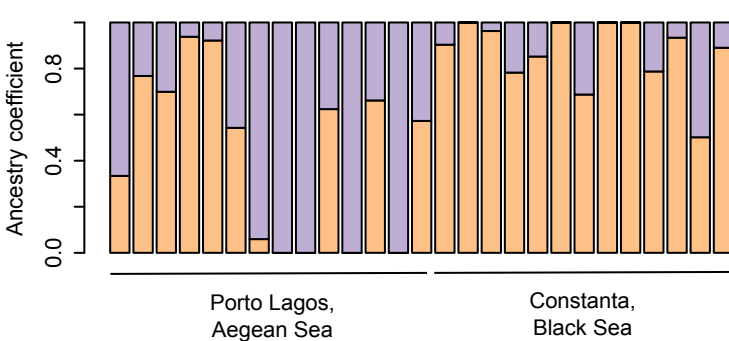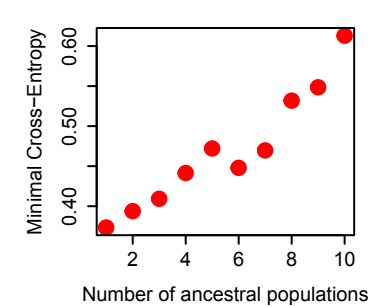

Supplement: Supplementary file 6 [file ECE3-9-4667-s006.pdf]

**A.**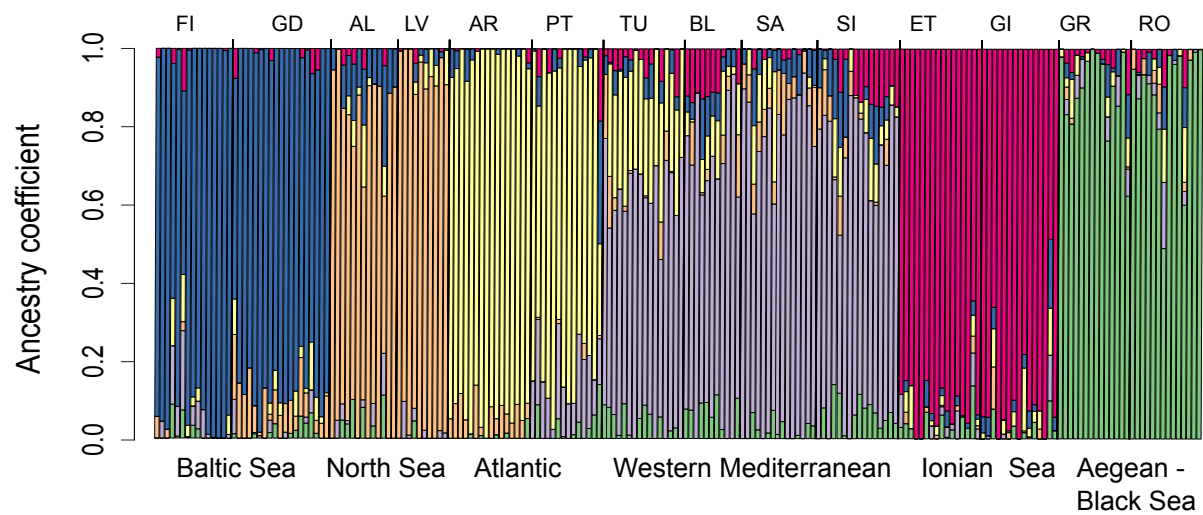**B.**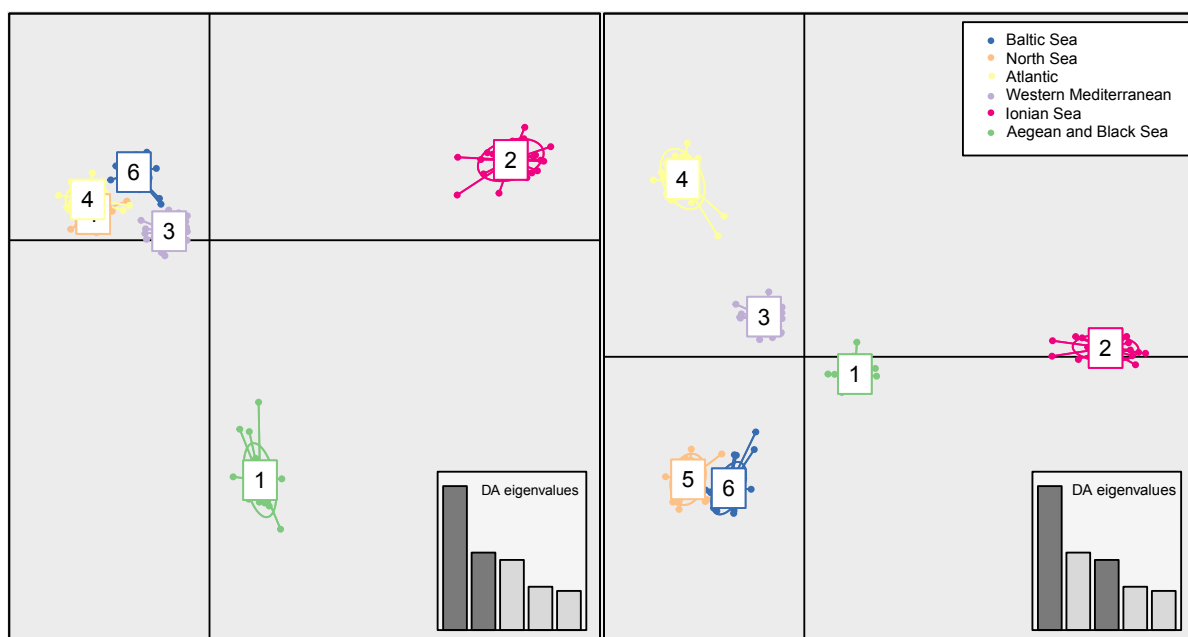

Supplement: Supplementary file 7 [file ECE3-9-4667-s007.pdf]
