# Supplementary material for: Next‐generation phylogeography of the cockle Cerastoderma glaucum: Highly heterogeneous genetic differentiation in a lagoon species
Source: Ecol Evol. 2019 Mar 27;9(8):4667–82. doi: 10.1002/ece3.5070 (PMC6476780; doi:10.1002/ece3.5070)
Supplement: Supplementary file 2 [file ECE3-9-4667-s002.pdf]

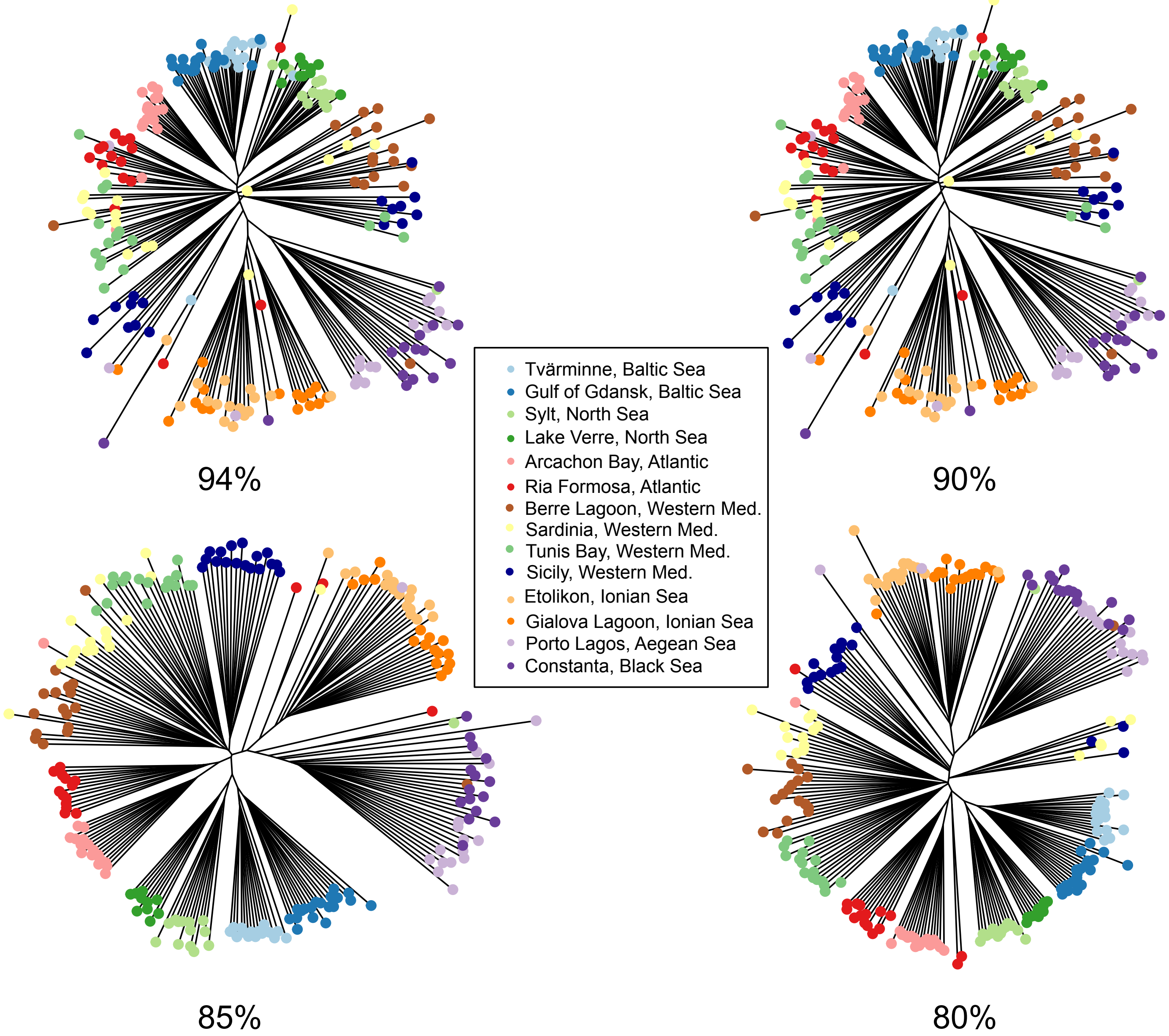
94%
90%
Tvärminne, Baltic Sea
Gulf of Gdansk, Baltic Sea
Sylt, North Sea
Lake Verre, North Sea
Arcachon Bay, Atlantic
Ria Formosa, Atlantic
Berre Lagoon, Western Med.
Sardinia, Western Med.
Tunis Bay, Western Med.
Sicily, Western Med.
Etolikon, Ionian Sea
Gialova Lagoon, Ionian Sea
Porto Lagos, Aegean Sea
Constanta, Black Sea
85%
80%
