# Supplementary material for: Next‐generation phylogeography of the cockle Cerastoderma glaucum: Highly heterogeneous genetic differentiation in a lagoon species
Source: Ecol Evol. 2019 Mar 27;9(8):4667–82. doi: 10.1002/ece3.5070 (PMC6476780; doi:10.1002/ece3.5070)
Supplement: Supplementary file 8 [file ECE3-9-4667-s008.docx]

**Table A1**. Pairwise *F*_ST_ values for *C. glaucum* population pairs

|  | **FI** | **GD** | **AL** | **LV** | **AR** | **PT** | **BL** | **SA** | **TU** | **SI** | **GI** | **ET** | **GR** |
| --- | --- | --- | --- | --- | --- | --- | --- | --- | --- | --- | --- | --- | --- |
| **FI** |  |  |  |  |  |  |  |  |  |  |  |  |  |
| **GD** | 0.096 |  |  |  |  |  |  |  |  |  |  |  |  |
| **AL** | 0.243 | 0.193 |  |  |  |  |  |  |  |  |  |  |  |
| **LV** | 0.247 | 0.200 | 0.166 |  |  |  |  |  |  |  |  |  |  |
| **AR** | 0.336 | 0.304 | 0.281 | 0.285 |  |  |  |  |  |  |  |  |  |
| **PT** | 0.302 | 0.281 | 0.291 | 0.275 | 0.181 |  |  |  |  |  |  |  |  |
| **BL** | 0.223 | 0.199 | 0.199 | 0.197 | 0.225 | 0.187 |  |  |  |  |  |  |  |
| **SA** | 0.283 | 0.263 | 0.253 | 0.267 | 0.270 | 0.208 | 0.081 |  |  |  |  |  |  |
| **TU** | 0.263 | 0.245 | 0.249 | 0.248 | 0.207 | 0.137 | 0.107 | 0.096 |  |  |  |  |  |
| **SI** | 0.248 | 0.244 | 0.238 | 0.234 | 0.245 | 0.192 | 0.094 | 0.112 | 0.101 |  |  |  |  |
| **GI** | 0.434 | 0.414 | 0.410 | 0.409 | 0.413 | 0.366 | 0.291 | 0.322 | 0.316 | 0.245 |  |  |  |
| **ET** | 0.403 | 0.384 | 0.378 | 0.381 | 0.384 | 0.335 | 0.254 | 0.286 | 0.284 | 0.210 | 0.062 |  |  |
| **GR** | 0.407 | 0.385 | 0.377 | 0.382 | 0.388 | 0.344 | 0.256 | 0.281 | 0.283 | 0.239 | 0.285 | 0.250 |  |
| **RO** | 0.412 | 0.390 | 0.386 | 0.393 | 0.397 | 0.351 | 0.262 | 0.287 | 0.290 | 0.244 | 0.297 | 0.258 | 0.030 |

Site code: **FI** - Tvärminne, Balic Sea; **GD** - Gulf of Gdansk, Balic Sea; **AL** - Sylt, North Sea; **LV -** Lake Veere, North Sea; **AR** - Arcachon Bay, Atlantic; **PT -** Ria Formosa, Atlantic; **BL** - Berre Lagoon, Western Mediterranean; **SA** - Sardinia, Western Mediterranean; **TU** - Tunis Bay, Western Mediterranean; **SI** - Siciny, Western Mediterranean; **GI** - Gialova Lagoon, Ionian Sea; **ET** - Etolikon, Ionian Sea; **GR** - Porto Lagos, Aegean Sea; **RO** - Constanta, Black Sea

**Table A2**. Pairwise *F*_ST_ values along with confidence intervals for *C. glaucum* population pairs calculated using bootstrapping across loci (10000 bootstrap replicates)

| Pop_1 | Pop_2 | Lower bound CI limit | Upper bound CI limit | *F*_st_ |
| --- | --- | --- | --- | --- |
| AL | AR | 0.260347 | 0.301235 | 0.280762 |
| AL | BL | 0.182353 | 0.216446 | 0.199469 |
| AL | ET | 0.359419 | 0.399819 | 0.379767 |
| AL | FI | 0.22119 | 0.26486 | 0.24307 |
| AL | GD | 0.173577 | 0.212413 | 0.192981 |
| AL | GI | 0.389314 | 0.429792 | 0.409888 |
| AL | GR | 0.355941 | 0.396866 | 0.376922 |
| AL | SI | 0.21954 | 0.255622 | 0.237603 |
| AL | LV | 0.146936 | 0.184565 | 0.165727 |
| AL | PT | 0.270608 | 0.311351 | 0.291222 |
| AL | RO | 0.364791 | 0.40624 | 0.386185 |
| AL | SA | 0.233146 | 0.272141 | 0.252737 |
| AL | TU | 0.230391 | 0.268253 | 0.249247 |
| AR | BL | 0.20672 | 0.2434 | 0.225296 |
| AR | ET | 0.364556 | 0.403751 | 0.384263 |
| AR | FI | 0.313632 | 0.358763 | 0.336181 |
| AR | GD | 0.282149 | 0.324442 | 0.303682 |
| AR | GI | 0.392933 | 0.43201 | 0.412902 |
| AR | GR | 0.367738 | 0.407753 | 0.388265 |
| AR | SI | 0.227626 | 0.262166 | 0.245082 |
| AR | LV | 0.263593 | 0.306468 | 0.285414 |
| AR | PT | 0.164731 | 0.19782 | 0.18139 |
| AR | RO | 0.376858 | 0.417315 | 0.397398 |
| AR | SA | 0.249486 | 0.290639 | 0.270222 |
| AR | TU | 0.188684 | 0.225769 | 0.20747 |
| BL | ET | 0.237913 | 0.26989 | 0.253969 |
| BL | FI | 0.206152 | 0.239519 | 0.222711 |
| BL | GD | 0.182021 | 0.214555 | 0.19853 |
| BL | GI | 0.274303 | 0.307324 | 0.290941 |
| BL | GR | 0.2391 | 0.272207 | 0.255771 |
| BL | SI | 0.084404 | 0.104418 | 0.094359 |
| BL | LV | 0.180413 | 0.21345 | 0.196714 |
| BL | PT | 0.171228 | 0.201669 | 0.186798 |
| BL | RO | 0.245603 | 0.278644 | 0.262083 |
| BL | SA | 0.069768 | 0.092054 | 0.080837 |
| BL | TU | 0.096436 | 0.11743 | 0.1069 |
| ET | FI | 0.382094 | 0.42354 | 0.403223 |
| ET | GD | 0.362958 | 0.403538 | 0.383655 |
| ET | GI | 0.055207 | 0.06959 | 0.062327 |
| ET | GR | 0.233998 | 0.266386 | 0.250428 |
| ET | SI | 0.194986 | 0.224129 | 0.209541 |
| ET | LV | 0.359203 | 0.401655 | 0.380796 |
| ET | PT | 0.315975 | 0.353615 | 0.334997 |
| ET | RO | 0.241334 | 0.274462 | 0.258187 |
| ET | SA | 0.268824 | 0.303152 | 0.285964 |
| ET | TU | 0.26704 | 0.300637 | 0.28408 |
| FI | GD | 0.082604 | 0.109096 | 0.095804 |
| FI | GI | 0.413112 | 0.453581 | 0.433859 |
| FI | GR | 0.385943 | 0.427034 | 0.406807 |
| FI | SI | 0.231583 | 0.264236 | 0.248149 |
| FI | LV | 0.225379 | 0.268567 | 0.247287 |
| FI | PT | 0.279963 | 0.323325 | 0.301728 |
| FI | RO | 0.39013 | 0.432779 | 0.411863 |
| FI | SA | 0.264816 | 0.301483 | 0.283379 |
| FI | TU | 0.246115 | 0.280493 | 0.263353 |
| GD | GI | 0.393343 | 0.433931 | 0.414106 |
| GD | GR | 0.363942 | 0.404832 | 0.38526 |
| GD | SI | 0.227062 | 0.259874 | 0.243708 |
| GD | LV | 0.181484 | 0.218733 | 0.200006 |
| GD | PT | 0.259672 | 0.300361 | 0.280516 |
| GD | RO | 0.368885 | 0.409921 | 0.390191 |
| GD | SA | 0.243722 | 0.281793 | 0.263185 |
| GD | TU | 0.227975 | 0.26182 | 0.244908 |
| GI | GR | 0.267704 | 0.301048 | 0.284746 |
| GI | SI | 0.229532 | 0.260064 | 0.244918 |
| GI | LV | 0.387449 | 0.429424 | 0.408765 |
| GI | PT | 0.347033 | 0.384661 | 0.366056 |
| GI | RO | 0.279942 | 0.314263 | 0.297255 |
| GI | SA | 0.303433 | 0.339659 | 0.321685 |
| GI | TU | 0.297852 | 0.333443 | 0.316006 |
| GR | SI | 0.222442 | 0.254748 | 0.238585 |
| GR | LV | 0.360231 | 0.40227 | 0.381784 |
| GR | PT | 0.324429 | 0.362464 | 0.34393 |
| GR | RO | 0.022662 | 0.037673 | 0.029839 |
| GR | SA | 0.263144 | 0.299546 | 0.281363 |
| GR | TU | 0.265999 | 0.300692 | 0.283164 |
| SI | LV | 0.216519 | 0.251142 | 0.233732 |
| SI | PT | 0.176029 | 0.208106 | 0.192199 |
| SI | RO | 0.227734 | 0.260315 | 0.24386 |
| SI | SA | 0.100302 | 0.124828 | 0.112441 |
| SI | TU | 0.090281 | 0.111606 | 0.100831 |
| LV | PT | 0.253202 | 0.296213 | 0.275427 |
| LV | RO | 0.370975 | 0.413677 | 0.392905 |
| LV | SA | 0.246532 | 0.286563 | 0.266662 |
| LV | TU | 0.228689 | 0.266382 | 0.247568 |
| PT | RO | 0.331727 | 0.369866 | 0.351141 |
| PT | SA | 0.190508 | 0.224924 | 0.207885 |
| PT | TU | 0.121797 | 0.152344 | 0.137096 |
| RO | SA | 0.268718 | 0.305644 | 0.287307 |
| RO | TU | 0.27196 | 0.307346 | 0.289677 |
| SA | TU | 0.084306 | 0.107265 | 0.095904 |

**Table A3.** Analysis of molecular variance testing the partitioning of genetic variation across populations and regions

| Analysis with six groups (Baltic, North Sea, Atlantic, Western Mediterranean, Ionian Sea, Aegean-Black Sea) | | | | | |
| --- | --- | --- | --- | --- | --- |
|  | df | | Variation (%) | Φ | *P* |
| Between groups | 5 | | 21.96 | 0.22 | 0.0001 |
| Between populations within groups | 8 | | 7.87 | 0.10 | 0.0001 |
| Between individuals within populations | 190 | | 13.61 | 0.19 | 0.0001 |
| Within individuals | 204 | | 56.55 | 0.43 | - |
| Analysis with five groups (Baltic-North Sea, Atlantic, Western Mediterranean, Ionian Sea, Aegean-Black Sea) | | | | | |
|  | | df | Variation (%) | Φ | *P* |
| Between groups | | 4 | 21.12 | 0.21 | 0.0001 |
| Between populations within groups | | 9 | 9.27 | 0.12 | 0.0001 |
| Between individuals within populations | | 190 | 13.50 | 0.19 | 0.0001 |
| Within individuals | | 204 | 56.11 | 0.43 | - |
|  | |  |  |  |  |
